# Protein Expression Modifications in Phage-Resistant Mutants of *Aeromonas salmonicida* after AS-A Phage Treatment

**DOI:** 10.3390/antibiotics7010021

**Published:** 2018-03-08

**Authors:** Catarina Moreirinha, Nádia Osório, Carla Pereira, Sara Simões, Ivonne Delgadillo, Adelaide Almeida

**Affiliations:** 1 Departament of Biology & CESAM, Campus Universitário de Santiago, Universidade de Aveiro, 3810-193 Aveiro, Portugal; anacatarinafernandes@gmail.com (C.M.); csgp@ua.pt (C.P.); 2 Escola Superior de Tecnologia da Saúde, Rua 5 de Outubro, SM Bispo. Instituto Politécnico de Coimbra, Apartado 7006, 3046-854 Coimbra, Portugal; nadia.osorio@estescoimbra.pt (N.O.); sarajmsimoes@hotmail.com (S.S.); 3 Departament of Chemistry, QOPNA, University of Aveiro, Campus Universitário de Santiago, 3810-193 Aveiro, Portugal; ivonne@ua.pt

**Keywords:** phage therapy, *Aeromonas salmonicida*, furunculosis, phage-resistant mutants, proteins, infrared spectroscopy

## Abstract

The occurrence of infections by pathogenic bacteria is one of the main sources of financial loss for the aquaculture industry. This problem often cannot be solved with antibiotic treatment or vaccination. Phage therapy seems to be an alternative environmentally-friendly strategy to control infections. Recognizing the cellular modifications that bacteriophage therapy may cause to the host is essential in order to confirm microbial inactivation, while understanding the mechanisms that drive the development of phage-resistant strains. The aim of this work was to detect cellular modifications that occur after phage AS-A treatment in *A. salmonicida*, an important fish pathogen. Phage-resistant and susceptible cells were subjected to five successive streak-plating steps and analysed with infrared spectroscopy, a fast and powerful tool for cell study. The spectral differences of both populations were investigated and compared with a phage sensitivity profile, obtained through the spot test and efficiency of plating. Changes in protein associated peaks were found, and these results were corroborated by 1-D electrophoresis of intracellular proteins analysis and by phage sensitivity profiles. Phage AS-A treatment before the first streaking-plate step clearly affected the intracellular proteins expression levels of phage-resistant clones, altering the expression of distinct proteins during the subsequent five successive streak-plating steps, making these clones recover and be phenotypically more similar to the sensitive cells.

## 1. Introduction

Aquaculture produces around 30% of the seafood for human consumption, being an increasingly important food fish source worldwide [1]. Generally, fish aquaculture is subjected to greater stress than wild conspecifics, which affects their natural immune system and often favours bacterial infection, especially during early life stages. This happens because of the high organic content and low concentration of dissolved oxygen often recorded in culture water, as well as the proximity of cultured individuals. Thus, opportunistic infections can easily emerge, causing significant economic losses to producers [2].

The Food and Agriculture Organization (FAO) and most aquaculture organizations recommend a decrease, or even the avoidance, of antibiotics in aquaculture, though they are still often used by the industry worldwide [1]. This can lead to the development of resistant bacteria and dispersal of antibiotic resistance in the environment, indirectly affecting bacterial species that are not associated with disease (non-target), allowing resistant strains to enter the human food chain [3,4]. 

Although vaccination is considered the best approach for the prevention of fish infections, it is practically impossible to employ during fish early life stages, due to their small size and low capacity to develop immunity [5,6]. Consequently, the development and application of innovative treatment technologies are demanded by the fish farming industry in order to increase the efficacy of aquaculture production, by lowering production costs and fish mortality, with reduced environmental impacts. 

*Aeromonas salmonicida*, the causative agent of furunculosis, is a significant fish pathogen in aquaculture. This disease causes high mortality and morbidity in a broad variety of fish, with important economic losses in aquaculture worldwide [7]. The chronic skin ulcers in weakened old fish make them unsuitable for human consumption [8]. The acute form is more common in juveniles and, usually, leads to septicaemia, being fatal in two to three days [9,10]. 

Phage therapy is an alternative approach to treat fish bacterial infections, being based on the use of bacteriophages (viruses that infect bacteria) to inactivate pathogenic bacteria. Compared to conventional methods such as antibiotics and vaccination, it presents several advantages: (a) phages are target specific; (b) serious or irreversible side effects of phage addition are not known; (c) phage therapy is an environmentally friendly strategy; (d) phages are resistant to various environmental conditions; (e) phage therapy is a flexible, fast and inexpensive technology [11,12]. Consequently, phage therapy appears to be a promising and environmentally friendly methodology to control bacterial infection. However, there are some studies reporting the development of phage-resistance by some bacteria [13,14,15,16]. This resistance may be due to the modification or loss of the bacterial cell surface receptors, blocking of the receptors by the bacterial extracellular matrix, production of modified restriction endonucleases that degrade the phage DNA, and inhibition of phage DNA penetration [17]. Additional causes for the development of resistance again bacteriophages are genetic mutations affecting phage receptors, restriction modification or abortive infection associated with the presence of clustered regularly interspaced short palindromic repeats (CRISPRs) in the bacterial genome [17,18]. Apart from genetic, resistance may also be phenotypic, which has been mostly disregarded in the literature [12,19,20]. It has been previously hypothesized that some of the reasons for phenotypic resistance may be: (i) induced, the products of phage-lysed bacteria result in a change in uninfected bacterial gene expression, thus reducing adsorption; (ii) intrinsic, reduced adsorption is due to a physiological or gene expression state that happens prior to the phage introduction; and (ii) dynamic, degradation or blocking of bacterial receptors by phage proteins released during cell lysis [19]. As very little is known about the effects of the phage infection in the bacterial cells, it is important to understand the inactivation mechanisms and the modifications that are induced by bacteriophages in the host cell, in order to obtain knowledge and a solution to the problem of phage-resistant bacteria.

Infrared spectroscopy (IR) has been a valuable method for detection and differentiation of microbial cells. It has also been successfully used to detect modification in proteins and lipids extracted from bacteria after exposure to a stress [21], and to study DNA structure [22]. Another advantage is the possibility of studying the whole cell, without the need to extract cellular components [23,24]. This methodology has already been used to discriminate phage-resistant from phage-susceptible bacteria [15]. The infrared absorbance spectrum represents a “fingerprint” that is characteristic of a chemical or biological substance. The main reasons for the wide acceptance of this method are the speed with which samples can be characterized with almost no handling, the flexibility of the equipment, the minimum sample amount required and the low cost of the analysis [25]. The analytical information from the spectra can be interpreted using a multivariate analysis that relates the spectra obtained with the properties of the object of study, thus facilitating data interpretation [26]. 

The main objective of this study was to understand the cellular modifications that occur in host targets after phage therapy, using the causative agent of furunculosis, *A. salmonicida*, and its specific phage AS-A as a model.

## 2. Results

### 2.1. Detection of Host Sensitivity to Phages after Phage Contact

**Figure 1:**
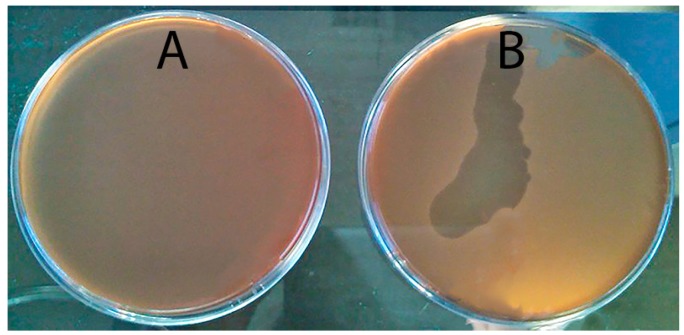
Spot test results using a phage-resistant mutant of phage AS-A and phage AS-A after first (**A**) and fifth streak-plating steps (**B**).

Firstly, five phage resistant colonies that grew inside a clear spot-test were selected for use in the subsequent steps. These colonies were smaller than the sensitive ones and took three times longer to appear on the petri plates. These colonies were subjected to five successive streak-plating steps. It was observed that the spot-tests were negative (Figure 1A) until the fourth streak-plating step, when the spot tests became positive (Figure 1B). However, efficiency of plating (EOP) results indicated that even after the fourth streak-plating step, phages neither form lysis plaques nor adsorb and replicate in the presence of the phage-resistant clones.

### 2.2. Infrared Spectroscopy of Whole Cells

The phage resistant clones from the five streaking-steps were analysed by IR spectroscopy in order to understand if there were any detectable differences in cellular components between these clones.

**Figure 2:**
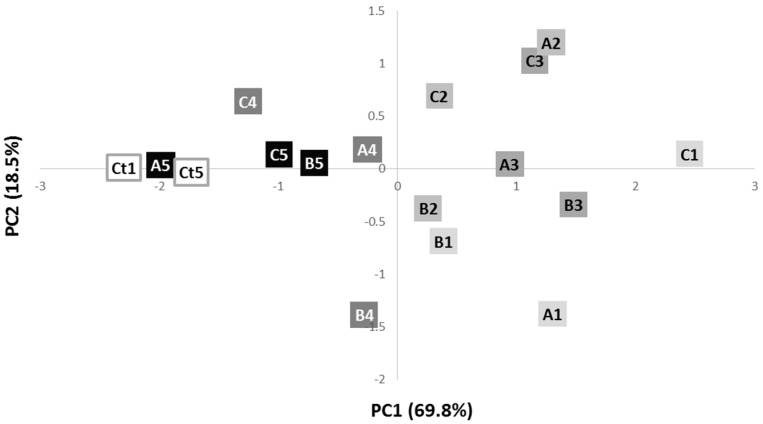
Scores scatter plot of the IR spectra of phage-resistant colonies A, B and C, along the 5 streak plating steps, and control phage sensitive colonies after 1 (Ct1) and 5 (Ct5) streaking steps. The letters correspond to the different colonies (A is colony A; B is colony B; C is colony C) and the numbers to the streaking-plate days (1 is day 1; 2 is day 2; 3 is day 3; 4 is day 4; 5 is day 5).

Principal component analysis (Figure 2) of the whole bacterial cells shows two distinct groups. It is visible a good discrimination between control phage-sensitive colonies and resistant colonies after the fourth and fifth streak-plating steps (negative PC1) and colonies from earlier streaking steps, corresponding to days 1, 2 and 3 (positive PC1).

**Figure 3:**
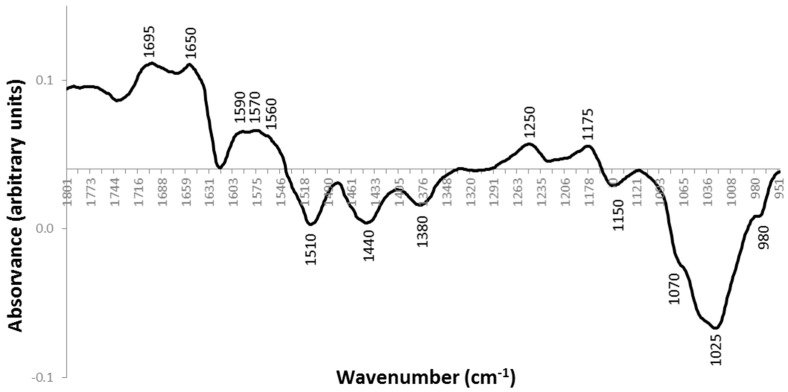
Loadings plot profile of PC1 corresponding to the IR spectra of the phage-resistant colonies A, B and C, along the 5 streak-plating steps, and control phage sensitive colonies.

Analysing the loadings plot profile (Figure 3), there are various peaks that are contributing to the distribution of the samples according to the principal component analysis (PCA). The samples that are located in negative PC1, that is, the later streaking days and the controls that are sensitive to phage are characterized by peaks at 1510 cm^−1^, 1440 cm^−1^, 1380 cm^−1^, 1150 cm^−1^, 1070 cm^−1^, 1025 cm^−1^ and 980 cm^−1^. The samples corresponding to the early streaking steps (1, 2 and 3), located in positive PC1, are characterized by peaks at 1695 cm^−1^, 1650 cm^−1^, 1590 cm^−1^, 1570 cm^−1^, 1560 cm^−1^, 1250 cm^−1^ and 1175 cm^−1^. Table 1 summarizes the infrared spectra peak assignments. It was found that the proteins were the most affected cellular component between phage-sensitive bacteria and phage-resistant bacteria. Taking into account these results, we decided to verify if there was also differential expression of the intracellular proteins in these cases. Phage-resistant clones of day 1, i.e., after one streak-plating step, and phage-resistant clones of day 5, i.e., after five streak-plating steps were chosen to perform protein analysis.

### 2.3. Differential Expression of the Proteins of the Phage-Resistant Clones (First Streak-Plating and Fifth Streak-Plating)

**Figure 4:**
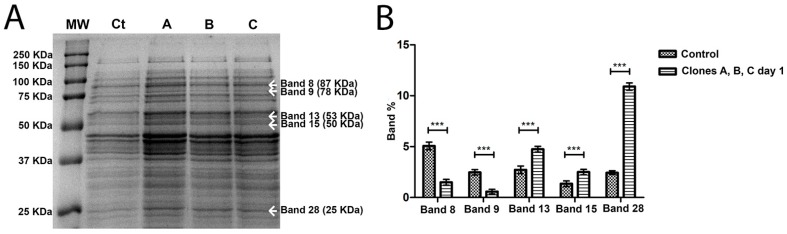
(**A**) SDS PAGE gel of the intracellular proteins of *A. salmonicida* on first streak-plating. MW, molecular weight marker; Ct, control phage-sensitive *A. salmonicida*; A is Colony A of the phage-resistant *A. salmonicida* mutant; B is Colony B of the phage-resistant *A. salmonicida* mutant; C is Colony C of the phage-resistant *A. salmonicida* mutant. The marked bands are the ones that showed differential expression between control and clones A, B and C. Band weight is expressed in kilodalton (KDa). (**B**) Differential expression of the bands, in percentage, comparing Control (phage-sensitive *A. salmonicida*) with clones A, B and C (phage-resistant *A. salmonicida*) after 1 streak-plating steps. *** *p* < 0.001.

**Figure 5:**
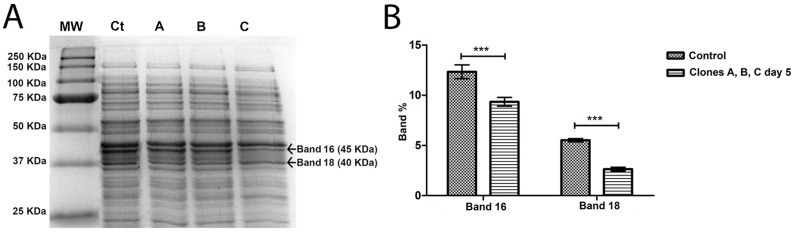
(**A**) SDS PAGE gel of the intracellular proteins of *A. salmonicida* on fifth streak plating. MW, molecular weight marker; Ct, control phage-sensitive *A. salmonicida*; A is Colony A of the phage-resistant *A. salmonicida* mutant; B is Colony B of the phage-resistant *A. salmonicida* mutant; C is Colony C of the phage-resistant *A. salmonicida* mutant. The marked bands are the ones that showed differential expression between control and clones A, B and C. Band weight is expressed in kilodalton (KDa). (**B**) Differential expression of the bands, in percentage, comparing Control (phage-sensitive *A. salmonicida*) with clones A, B and C (phage-resistant *A. salmonicida*) after 5 streak-plating steps. *** *p* < 0.001.

In order to try to understand why phage-sensitive bacteria (control) and clones after five streak-plating steps were different from clones after one streak-plating step, 1D SDS-PAGE gels were performed, comparing control and first streak-plating clones (Figure 4A), and comparing control and fifth streak-plating clones (Figure 5A).

In total, 39 bands were detected and compared, in the control and *A. salmonicida* clones, both in first streak-plating and fifth streak-plating clones. When compared to the control, the bands that were significantly differentially expressed on first streak-plating clones were bands 8, 9, 13, 15 and 28 (Figure 4B). The expression patterns of the bands 8 and 9 in *A. salmonicida* first streak-plating clones tend to be less when compared to the control. However, the 13, 15 and 28 bands tend to have an increased expression compared to the control (Figure 4B). On fifth streak-plating clones, the differentially expressed bands were band 16 and 18 (Figure 5B). All of the bands with differential expression decreased between the control and fifth streak-plating clones.

By using the homology of the molecular weight of the bands with differential expression, consulting the databases referred to in the Section 4, presumptive identification of the proteins was made (Table 2). 

## 3. Discussion

The emergence of phage-resistant mutants during phage infection has been reported in many studies [12,37,38,39,40,41], but the mechanisms of bacteria resistance to phages are not yet completely understood. A previous study by our group [41] showed that the agent of furunculosis can be efficiently inactivated by the phage AS-A (reduction of 4 Log CFU·mL^−1^ after 8 h of treatment). However, some bacteria survived the infection by the phage due to the development of phage-resistance [41]. Nevertheless, the frequency of resistance, with a value of 2.24 × 10^−4^ Log CFU·mL^−1^, was limited as already reported in previous studies [14,42,43].

So, in our previous study [41] we verified that although a specific phage against the agent of furunculosis can efficiently control the bacterial growth, some phage-resistant bacteria emerge after treatment. In the present study, we observed that the resistant colonies after the fourth and fifth streak-plating steps are clearly distinct from those of the earlier streaking steps (steps 1, 2 and 3). A significant modification in the expression of intracellular proteins was observed when compared with the phage-sensitive bacteria. Moreover, these modifications affect distinct proteins after the first and the fifth streak-plating steps, allowing “lysis from without” (positive spot test) after the forth streak-plating step, contrary to that observed for bacteria from the first, second and third streak-plating steps.

It has been stated in the literature that resistance to phages can be overcome by the phage itself because it evolves along with the host [44]. Moreover, it has also been asserted that resistance to phages entails great costs to the bacteria [45]. In fact, as observed for other phages, colonies of AS-A phage-resistant mutants were smaller than colonies formed by the non-phage added control [14]. These results suggest that the remaining bacterial mutants (forming small size colonies) maintained their viability in the presence of phages but their phenotypes were affected. The decrease in the bacterial size after phage exposure could be a fitness cost, which might contribute to their elimination from the environment faster than their wild-type parents. 

In this study, as already observed for other phages [15,16], it was detected that phage-resistant bacteria also mutate after successive streak-plating steps. Although the spot tests showed negative results until the fourth streak-plating step, at the fourth and fifth steps, the spot test was positive, as also observed in other studies [15,16]. These results were confirmed by infrared spectroscopy data of the whole cells. Infrared spectroscopy results show that the spectra obtained from the fourth and fifth streak-plating colonies are similar to ones from phage-sensitive control colonies, suggesting that these colonies are more similar to control phage-sensitive bacteria than the colonies from streak-plating steps 1, 2 and 3. It seems that the resistant bacteria somehow “recovered”, being more similar to control bacterial populations, which are sensitive to the phage infection. The infrared peaks that contributed to these results were found to be especially associated with proteins. Taking this into account, we focused further studies on protein analysis with 1D SDS PAGE gels.

Regarding the presumptively identified proteins with differential expression on first streak-plating phage-resistant clones, a decrease in band 8 is noticeable when compared to the control, being the band associated with a phage transcriptional protein with regulation function in the transcription of phage genes [46]. This may be a response by the bacteria to the viral infection, preventing the transcription of the viral genome. Similarly, the expression of the protein corresponding to band 9 in first streak-plating clones decreased when compared to the control. This protein, phage-shock B protein, is involved in a regulation system that responds to aggression, habitually to phage secretins, promoting the defensive response of the bacteria [47]. This protein has been previously detected in the response of other bacteria, however, this response mechanism is not yet completely understood [47,48]. In our case, this protein is less expressed in the phage-resistant clones, which seems contradictory. Nevertheless, it was stated that bacteria synthesise phage shock proteins after being infected with phage, that, in the case of the resistant clones could not happen [49]. Contrarily, the protein associated to band 13, TatA, increased in *A. salmonicida* first streak-plating clones. This protein belongs to the Tat system (twin-arginine translocation) which is responsible for the transport of various substances at the membrane level, against the concentration gradient of the cytoplasm to the extracellular space, namely proteins, being associated with the bacterial pathogenicity in the secretion of virulence factors [50,51]. This increase suggests that these first streak-plating clones could be more virulent than control bacteria. However, some studies have shown that phage-resistant clones are less pathogenic than phage sensitive bacteria [20,52]. This suggests that the increase in the expression of this protein could be associated with other mechanisms not related with pathogenicity. 

Regarding the proteins with differential expression on phage-resistant clones in the fifth streak-plating, that have a positive spot-test, band 16 suggests the expression of a transposase that is decreased in these clones when compared to control phage-sensitive bacteria. These type of enzymes facilitates the transference of the genetic material between organisms [53]. The bacteria may have decreased the expression of this protein as a defence mechanism in order to prevent the phage replication. Band 18, corresponds to a toxin-antitoxin system, which is implied in the maintenance of plasmids, stress regulation and adaptation, as well as in growth control and programmed cellular death [54,55]. This system requires the dual activity of a toxin and an antagonistic antitoxin [56]. A decrease in this band in the clones of the fifth streak-plating was found when compared to the control. As this protein decreased in this study, this suggests that in the fifth streak-plating clones, the stress caused by the phage decreased. In fact, the efficiency of plating (EOP) results indicate that the fifth streak-plating clones do not replicate the phage. Other authors [57] have obtained similar results, designating this situation by “lysis from without”. The spot test lysis when the phage is not replicated by the host (EOP is zero) has been described as a plausible mechanism which happens when an overload of phage simultaneously infects a bacterium leading to lysis, either from the action of phage lysins or from rapid depletion of the cell resources [58]. As in the spot test the same volume of phage suspension was used and lysis was only observed for the clones of the fourth and fifth streak-plating, so, the hypothesis of rapid depletion of the cells resources does not seems plausible. As stated before, the lysis can be due to the presence of phage lysins. However, it is difficult to understand why the spot test was only positive for the clones of the fourth and fifth streak-plating and not for the clones of the first, second and third streak-plating. However, modifications in the bacterial proteins along the successive streak-plating could allow the clones to recover the sensitivity to the phage lysins. This is in agreement with the infrared spectroscopy results which showed that the fourth and fifth streak-plating clones were similar to the phage-sensitive bacteria (control), but clearly different from those of the first, second and third streak-plating steps. In order to test this hypothesis, further studies are needed. It would be interesting, for example, to try to correlate IR spectra with the regaining of sensitivity to phage lysins to extract more information from the spectra.

We noticed that the different analysed clones present significant modifications in intracellular proteins related to phage infection, both in the first and fifth streak-plating steps. However, there are more proteins that are differentially expressed in clones of the first streak-plating than in clones of the fifth streak-plating, which is in accordance with infrared spectroscopy results. The fact that the phage-sensitive control bacteria have infrared spectra that are more similar to the fourth and fifth streak-plating clones may be because the cellular envelope, used by the phages to infect the bacteria, became more similar in these cases. This may be related to the fact that the spot test turns positive again for the fourth and fifth streak-plating clones, which might be due to phenotypical similarities in the cell envelope. In our study, phenotypic resistance may have been acquired by phage-resistant cells, showing less pronounced cell modifications than genetic resistance, which would be more definitive. In order to better understand this, more experiments should be done, such as serial dilution spot-tests and EOP tests with varying multiplicity of infection (MOI). In order to confirm these results, the presumptively identified proteins and the non-identified proteins that show differential expression between the clones should be confirmed/identified by methods such as mass spectrometry. In future experiments, it would also be interesting to include the whole cell proteins, which would provide more information. Moreover, since there are some indications of which proteins seem to alter their expression, molecular assays using specific primers for these proteins would be a reliable method to use in order to explore and elucidate the whole process of the clone expression pattern.

## 4. Materials and Methods

### 4.1. Bacteria and Phage

The bacteria *A. salmonicida* CECT 894 was used in this study. Fresh plate bacterial cultures were maintained in solid Tryptic Soy Agar medium (TSA; Liofilchem, Roseto degli Abruzzi, Italy) at 4 °C. Before each assay, one isolated colony was aseptically transferred to 10 mL of Tryptic Soy Broth medium (TSB; Liofilchem, Roseto degli Abruzzi, Italy) and was grown overnight at 25 °C. An aliquot of this culture (100 μL) was aseptically transferred to 10 mL of fresh TSB medium (Liofilchem, Roseto degli Abruzzi, Italy) and grown overnight at 25 °C to reach an optical density (O.D. 600) of 0.8, corresponding to about 10^9^ cells·mL^−1^.

Phage AS-A was isolated from sewage water from a lift station of the sewage network of Aveiro, Portugal (station EEIS9 of SIMRIA Multi Sanitation System of Ria de Aveiro) using *A. salmonicida* as host, according to [41]. The phage stocks were stored at 4 °C and 1% chloroform (final volume) (Scharlau, Sentmenat, Spain) was added. The phage suspension titre was determined by the double-layer agar method using TSA (Liofilchem, Roseto degli Abruzzi, Italy) as the culture medium [59]. The plates were incubated at 25 °C for 12 h and the number of lysis plaques was counted. The results were expressed as plaque forming units per millilitre (PFU·mL^−1^).

### 4.2. Isolation of A. salmonicida Phage-Resistant Mutants

Only bacterial colonies that were resistant to the phage were used (bacteria that developed inside phage plates). For this, bacteria *A. salmonicida* and phage AS-A were plated by the double layer agar method and the plates were incubated for 24 h at 25 °C. After that, several colonies that grew inside the phage plates, thus, resistant to phage infection, were visible. Three individualized colonies (A, B and C) were chosen and used in the subsequent assays.

### 4.3. Detection of Bacteria Sensitivity to the Phage after One Cycle of Phage Contact

The phage resistant colonies obtained in Section 4.2 were used. The colonies were inoculated in TSB medium for 24 h at 25 °C. After that, the culture was used to perform a spot test and was also plated in TSA medium. This procedure was done 4 more times, making a total of 5 streak plating steps. This procedure was done for the 3 selected colonies.

### 4.4. Efficiency of Plating (EOP)

The efficiency of plating was determined for bacteria that shown positive spot tests (clear lysis area), i.e., for the bacteria from the fourth and fifth streak-plating steps, according to Pereira et al. [15] using the double-agar method [59]. The EOP was calculated (average PFU on target bacteria/average PFU on host bacteria), three independent assays were performed.

### 4.5. Phage Adsorption

The determination of phage adsorption was performed according to Pereira et al. [15]. Briefly, ten microliters of phage suspension of about 10^6^ PFU/mL were added to 10 mL of *A. salmonicida* culture of about 10^9^ CFU/mL (corresponding to an optical density (600 nm) of 0.8) [60] and incubated at 25 °C. Aliquots of this culture were collected after 0, 5, 10, 15, 20, 25, 30, 40, 50, 60 and 70 min of incubation and chloroform was added to a final concentration of 1%. The mixture was centrifuged at 12,000× *g* for 5 min, after that the supernatants were filtered using 0.2 µL membranes (Millipore, Bedford, VA, USA). The filtrates containing unadsorbed phages were then diluted and titrated. The plates were then incubated at 25 °C and observed after 8 h for plaque formation. The values were calculated as the decrease of phage titre in supernatant (percentage) compared with time zero. Three independent assays were performed.

### 4.6. Infrared Spectroscopy

In order to access the spectral differences of sensitive *A. salmonicida* colonies and phage resistant mutant colonies, mid-infrared spectroscopy was used, as it was previously described [15,24]. They were used for the *A. salmonicida* phage resistant colonies A, B and C (from Section 4.3).

To analyse the whole cells, colonies A, B and C were analysed during the 5 days of streaking (Section 4.3), as well as control sensitive colonies Ct1 and Ct5 (after the 1 and 5 streak plating steps). The colonies were collected with a loop and placed in the crystal of a horizontal single reflection ATR accessory. The colonies were gently air dried and the spectra were acquired.

Spectra were done in a MIR (Bruker ALPHA FTIR spectrometer, Germany) with a resolution of 4 cm^−1^ and 32 scans, in the infrared region (4000 to 600 cm^−1^). At least 5 replicate spectra were performed for each colony. Mid-infrared spectra were obtained in OPUS format (OPUS 6.5, Bruker, Germany) and transferred via JCAMP.DX format for use in a house-developed data analysis software (CATS build 97). The spectra were SNV (standard normal deviate) corrected previous to multivariate analysis. Principal component analysis (PCA) was done in order to find the major sources of variability in the spectra and to detect groups.

### 4.7. Extraction and Quantification of Intracellular Proteins from Phage-Sensitive and Phage-Resistant Bacteria

The proteins extracts were obtained from the growth until the late exponential phase of the strains (OD 0.9 at 550 nm) in Luria Bertani Broth (Merck, Darmstadt, Germany). The cells were separated from the supernatant by centrifugation at 8000× *g* for 10 min at 4 °C. The protein extractions were made in three independent experiments per each strain and the protein quantification was performed in triplicate.

The cell pellets were washed three times in 10 mM phosphate buffered saline pH 7.4. After that they were resuspended in 1 mL of lysis and protein solubilisation buffer solution (7 M urea, 2 M thiourea, 4% cholamidopropyl dimethylammonio-1-propanesulfonate (CHAPS), 30 mM Tris base, pH 8.5). Crude cell-free extracts were obtained by sonication in ice to minimize protein damage, during a 2 min period, using a 30% duty cycle, 2 s pulses with intervening periods of 3 s. The intracellular protein solution was incubated with 1 mg·mL^−1^ of Dnase I (GE Healthcare, Uppsala, Sweden) and 10 mM of protease inhibitor mix (GE Healthcare, Uppsala, Sweden) for 1 h at 15 °C. The final solution was collected by centrifugation at 20,000× *g* for 40 min at 4 °C and then, the protein concentration was measured using the 2-D Quant Kit (GE Healthcare, Uppsala, Sweden), following the manufacturer’s instructions. The procedure was performed in triplicate.

### 4.8. Protein Separation by 1-D Electrophoresis

Proteins were separated by 12.5% SDS-PAGE [61], in a Mini-PROTEAN 3 Cell (Bio-Rad, Hercules, CA, USA), for 50 min at 150 V. 5 µg/mL of each protein sample were used in this assay. Proteins were visualized by colloidal Coomassie Brilliant BlueG-250 (CBB) staining [62]. Gel images were acquired using the Gel DocTM XR+ (Bio-Rad, Hercules, CA, USA). The comparative analysis of the acquired images was performed in Image Lab v3.0 software (Biorad, Hercules, CA, USA) and based on the optical density measurement of each band. To minimize possible differences in the quantity of the proteins loaded, the results were normalized and expressed as a band percentage, resulting from the value of the optical density of a given band in the total of the bands per lane × 100. The comparison of the differential expression of the intracellular proteins of the different tested *A. salmonicida* clones in the different analysis times was made through a two-way ANOVA, using GraphPad Prism software v7 (USA). The differences were considered statistically significant when *p* < 0.05. 

### 4.9. Presumptive Identification of the Proteins in Differentially Expressed Bands

The molecular weight of the bands that were differentially expressed between control and *A. salmonicida* clones on day 1 and between control and day 5, using the databases UniProtKB (www.uniprot.org) and NCBI (www.ncbi.nlm.nih.gov/pubmed) allowed us to presumptively identify the proteins and their respective function, based on the deposited genome of *Aeromonas salmonicida* A449. 

## 5. Conclusions

A single cycle of phage treatment causes a significant modification in the expression of intracellular proteins of phage-resistant bacterial clones relative to the phage sensitive bacteria, but after successive streaking-plate steps these clones recover and are phenotypically more similar to the sensitive cells. Taking this information into account, this study paves the way for future experiments in order to better understand the bacterial resistance mechanisms to phages.

## Figures and Tables

**Table 1:** Peaks/regions assignments (wavenumber) from principal component analysis (PCA) loadings plot profile of spectra from colonies of *A. salmonicida* sensitive and resistant to phage AS-A.

PC1 − (cm^−1^)	PC1 + (cm^−1^)	Assignment	Reference
	1695	Amide I—proteins (β-sheet)	[27]
	1650	Amide I—proteins (α-helix)	[23,27]
	1590, 1570, 1560	Amide II—proteins	[27]
1510		Amide II—proteins	[27]
1440		CH_3_ bending—proteins (methyl groups)	[28]
1380		COO^−^—acids and methyl groups from proteins/CO bonds or deformation of C-H or N-H bonds of proteins	[28,29]
	1250	Amide III—proteins/PO_2_^−^—phospholipids	[30,31]
	1175	C-O—proteins and glycomaterials	[32,33]
1150		C-O carbohydrates	[33]
1070		PO_2_^−^—nucleotides	[34]
1025		Carbohydrates	[35]
980		OCH_3_—polysaccharides	[36]

**Table 2:** Presumptive band identification of the 1-D electrophoresis gel of intracellular proteins, associated proteins and their molecular functions.

Band	MW (KDa)	Protein/Gene	Molecular Function
Band 8	87	Phage transcriptional protein (ASA_3866)	Interacts selectively and non-covalently with the DNA with a specific nucleotide composition or with a specific sequence motif or type of DNA.
Band 9	78	Phage shock protein B (pspB, ASA_2424)	Response of the bacteria to a variety of stimuli, including phage infection. It is involved in bacterial protection mechanisms.
Band 13	53	Sec-independent protein translocase proteinTatA (tatA, ASA_3970)	Biological process: controlled liberation of proteins from a cell.
Band 15	50	ASA_P5G151	Unknown function.
Band 16	45	Transposase (VO70_17345, VO70_21745)	Facilitates the transference of genetic material between organisms.
Band 18	40	Toxin-antitoxin system, toxin component (VO68_18510, VO70_09250)	Plasmid maintenance, stress regulation and adaptation, growth control and programmed cellular death.
Band 28	25	Q70WF0, Q70WF0_AERSA	Unknown function.
